# Analysis of Cyberincivility in Posts by Health Professions Students: Descriptive Twitter Data Mining Study

**DOI:** 10.2196/28805

**Published:** 2021-05-13

**Authors:** Jennie C De Gagne, Eunji Cho, Sandra S Yamane, Haesu Jin, Jeehae D Nam, Dukyoo Jung

**Affiliations:** 1 School of Nursing, Duke University Durham, NC United States; 2 School of Nursing, Vanderbilt University Nashville, TN United States; 3 Department of Nursing, Catawba College Salisbury, NC United States; 4 Division of Hematologic Malignancies and Cellular Therapy, Duke University Medical Center Durham, NC United States; 5 Global Health Institute, Duke University Durham, NC United States; 6 College of Nursing, Ewha Womans University Seoul Republic of Korea

**Keywords:** cyberincivility, digital professionalism, health professions students, social media, social networking sites, Twitter

## Abstract

**Background:**

Health professions students use social media to communicate with other students and health professionals, discuss career plans or coursework, and share the results of research projects or new information. These platforms allow students to share thoughts and perceptions that are not disclosed in formal education settings. Twitter provides an excellent window through which health professions educators can observe students’ sociocultural and learning needs. However, despite its merits, cyberincivility on Twitter among health professions students has been reported. *Cyber* means using electronic technologies, and *incivility* is a general term for bad manners. As such, *cyberincivility* refers to any act of disrespectful, insensitive, or disruptive behavior in an electronic environment.

**Objective:**

This study aims to describe the characteristics and instances of cyberincivility posted on Twitter by self-identified health professions students. A further objective of the study is to analyze the prevalence of tweets perceived as inappropriate or potentially objectionable while describing patterns and differences in the instances of cyberincivility posted by those users.

**Methods:**

We used a cross-sectional descriptive Twitter data mining method to collect quantitative and qualitative data from August 2019 to February 2020. The sample was taken from users who self-identified as health professions students (eg, medicine, nursing, dental, pharmacy, physician assistant, and physical therapy) in their user description. Data management and analysis were performed with a combination of SAS 9.4 for descriptive and inferential statistics, including logistic regression, and NVivo 12 for descriptive patterns of textual data.

**Results:**

We analyzed 20 of the most recent tweets for each account (N=12,820). A total of 639 user accounts were analyzed for quantitative analysis, including 280 (43.8%) medicine students and 329 (51.5%) nursing students in 22 countries: the United States (287/639, 44.9%), the United Kingdom (197/639, 30.8%), unknown countries (104/639, 16.3%), and 19 other countries (51/639, 8.0%). Of the 639 accounts, 193 (30.2%) were coded as having instances of cyberincivility. Of these, 61.7% (119/193), 32.6% (63/193), and 5.7% (11/193) belonged to students in nursing, medicine, and other disciplines, respectively. Among 502 instances of cyberincivility identified from 641 qualitative analysis samples, the largest categories were profanity and product promotion. Several aggressive or biased comments toward other users, politicians, or certain groups of people were also found.

**Conclusions:**

Cyberincivility is a multifaceted phenomenon that must be considered in its complexity if health professions students are to embrace a culture of mutual respect and collaboration. Students’ perceptions and reports of their Twitter experiences offer insights into behavior on the web and the evolving role of cyberspace, and potentially problematic posts provide opportunities for teaching digital professionalism. Our study indicates that there is a continued need to provide students with guidance and training regarding the importance of maintaining a professional persona on the web.

## Introduction

### Background

Over the past decades, social networking services have significantly improved communication and connection for millions of people worldwide. Twitter has been a particularly popular social networking platform since its launch in 2006 and currently has more than 330 million active users per month [1]. This platform enables users to post a short message with images or videos, exchange ideas or information with other users, and customize their information streams via a unique subscribing function (ie, following) [1]. The microblogging feature of Twitter allows users to share their thoughts within a limited number of characters, thus helping them to reorganize and polish their ideas concisely [2,3]. Owing to its ubiquitous nature, simplicity, and user connectivity, Twitter is widely used for a variety of purposes.

### Twitter and Health Professions

A growing body of research has identified Twitter as a useful tool for health care provider development [4,5]. Health care providers and health professions students use Twitter in various ways, including for intraprofessional and interprofessional mentoring and networking [6-8], knowledge development and discussion [9], idea and information sharing [10], teaching and learning [11,12], and contacting or communicating with patient groups [5,13,14]. Twitter is well positioned as a creative and convenient tool to help health care providers and health professions students develop skills beyond traditional boundaries [15].

Despite its advantages, previous studies on social media, including Twitter, have identified potential problems that may arise from misuse and misinterpretation. Health care professionals are among the sources of health-related information most trusted by the public [16]. Although students are not yet licensed experts, by sharing tweets while disclosing their identities as health professions students, they can earn public trust; conversely, their improper use of Twitter can have unexpected consequences. For instance, tweets perceived as misleading or lacking in sensitivity may cause the information conveyed to be perceived as inaccurate or may unintentionally offend some audiences, and such tweets can be preserved permanently [17].

Health care providers and health professions students can invade patients’ privacy by disclosing their personal information on Twitter or by sharing detailed clinical scenarios that the patients or their acquaintances can easily recognize [18]. Moreover, by displaying profanity, offensive language, aggression toward other health professionals, product promotion, violence, or any violation of patient confidentiality on Twitter, they could damage their reputation or lose public confidence [19-21]. Such misuses of Twitter can undermine its potential benefits, create misconceptions about health care professionals, and affect the privacy of health care providers and their colleagues and patients.

To maximize the benefits of Twitter use by health professions students, it is essential to promote cybercivility, or behavior in an electronic environment that reflects the norms and mutual respect that characterize the professional culture to which users belong and the society in which they live, learn, and work. In contrast to cybercivility, cyberincivility is defined as “direct and indirect interpersonal violation involving disrespectful, insensitive, or disruptive behavior of an individual in an electronic environment that interferes with another person’s personal, professional, or social well-being, as well as one’s learning” [22]. An understanding of the prevalence and properties of cyberincivility among health professions students can provide the foundational knowledge needed to develop instructional strategies and administrative guidelines regarding the use of social networking services to promote and maintain cybercivility in health professions education.

### Research Aim

This study aims to describe the characteristics and instances of cyberincivility posted on Twitter by self-identified health professions students. The specific objectives were to (1) analyze the prevalence of tweets that could be perceived as inappropriate or potentially objectionable for a health professions student and (2) describe the patterns and differences in instances of cyberincivility posted by those users.

## Methods

### Design and Sample

We used a cross-sectional Twitter data mining method to collect quantitative and qualitative data from August 2019 to February 2020. The sample was taken from health professions students in various disciplines, including medicine, nursing, dental, pharmacy, physician assistant, and physical therapy. We included only tweets written in English by users who self-identified as health professions students on their user description, but we did not limit the geographic location. cross-sectional Twitter data mining method

### Ethical Considerations

This study was reviewed and declared exempt by the institutional review board of Duke University (Pro00106123). To protect users’ privacy and their digital rights, we deidentified all identifiable personal information (eg, name, user identification, location, and affiliation) after data analysis. We also paraphrased all quotes presented as examples to prevent backtracking while maintaining their original meanings. Only data relevant to the purpose of this study were collected, and a secure, shared drive was used to store and manage all research data.

### Data Collection: Eligible Twitter Account List Development

Initially, we identified potential user accounts by searching for 50 hashtags (Textbox 1) through the desktop version of BirdIQ v1.6 [23], a cross-platform data extraction program tailored to Twitter queries using preselected hashtags. The search results were returned in a multitabbed Microsoft Excel [24] workbook that included tweeting accounts.

The search terms (Textbox 1) allowed us to compile original tweets that were written in English and contained a designated hashtag over a given period (ie, August 28, 2019, to September 25, 2019). We set the time interval to 1 week and ran the BirdIQ program once a week on the same day of the week and at the same time. As a result of this process, 12,360 tweets containing one or more of the 50 hashtags were collected over 5 weeks. After removing duplicates, the remaining 10,267 tweets were linked to 5671 accounts. We removed 1556 duplicates and excluded accounts based on the inclusion and exclusion criteria summarized in Textbox 2.

**Hashtag list.**

**Medical students:**

- #medicalstudent; #medschool; #medicalschool; #usmleprep; #usmlepreparation; #usmlexam; #usml; #futuredoctor; #medicalcollege; #medschoolthings; #medstudenttwitter; #premed; #medstudentlife; #medstudentblog; #lifeofamedstudent; #medical_student

**Nursing students:**

- #studentnurse; #nursingstudentproblems; #nursingschool; #nclexrnexam; #adnstudent; #bsnstudent; #msnstudent; #dnpstudent; #futurebsnrn; #futurern; #futurenurse; #futurenp; #futurenursepractitioner

**Students in other disciplines (dental, pharmacy, physician assistant, and physical therapy):**

- #dentalschool; #dentalstudent; #nbde; #futuredentist; #physicianassistantstudent; #PAschool; #futurePA; #PANCE; #pharmacystudent; #futurepharmacist; #pharmacyschool; #NAPLEX; #futurehealthcareprovider; #futurehealthprofessional; #healthstudent; #health_student; #futurephysicaltherapist; #futurePT; #PTstudent

**A search string example:**

- #nursingstudent -filter:retweets lang:en since:2019-9-17 until:2019-9-23.

**Account inclusion and exclusion criteria.**

**Account inclusion criteria:**

- Belongs to a student identified as a current health professions student (ie, medicine, nursing, dental, physician assistant, and physical therapy) on the user description
- Is written primarily in English
- Has more than 100 followers at the time of data collection
- Has more than 50 tweets written at the time of data collection
- Is open to public

**Account exclusion criteria:**

- Belongs to a postlicensure professional in clinical clerkship
- Belongs to a student not self-identified as such on the user description
- Belongs to a premed, prenursing, or research-only PhD student
- Suspended or locked over the course of data collection
- Is institutional, with an aim to provide information, education, or commercial advertisements to health professions students
- Has over 70% of tweets not written in English

**Figure 1:**
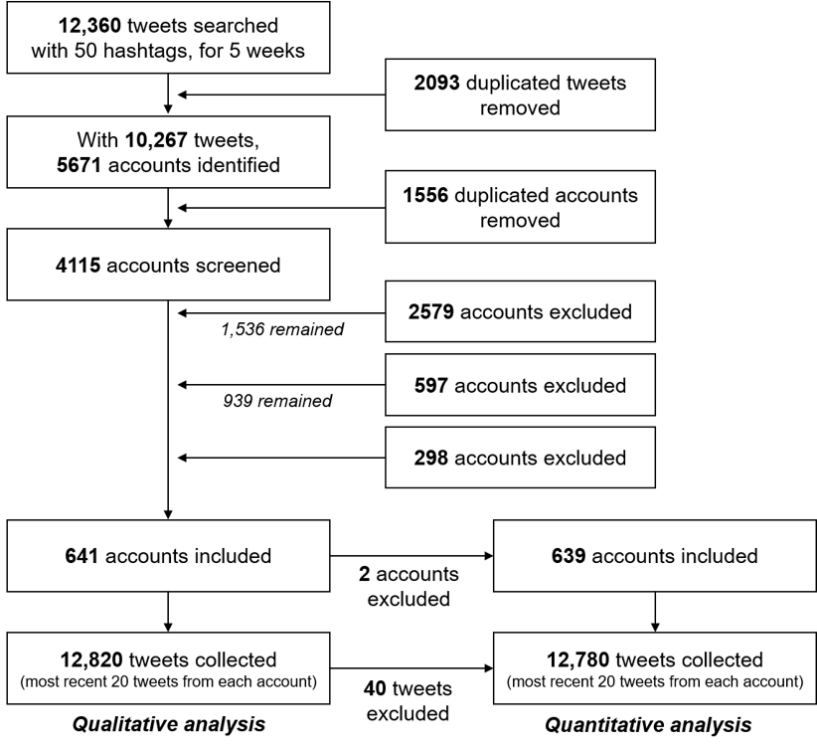
A flow diagram to depict data mining and sampling procedures. PA: physician assistant; PT: physical therapy.

**Figure 2:**
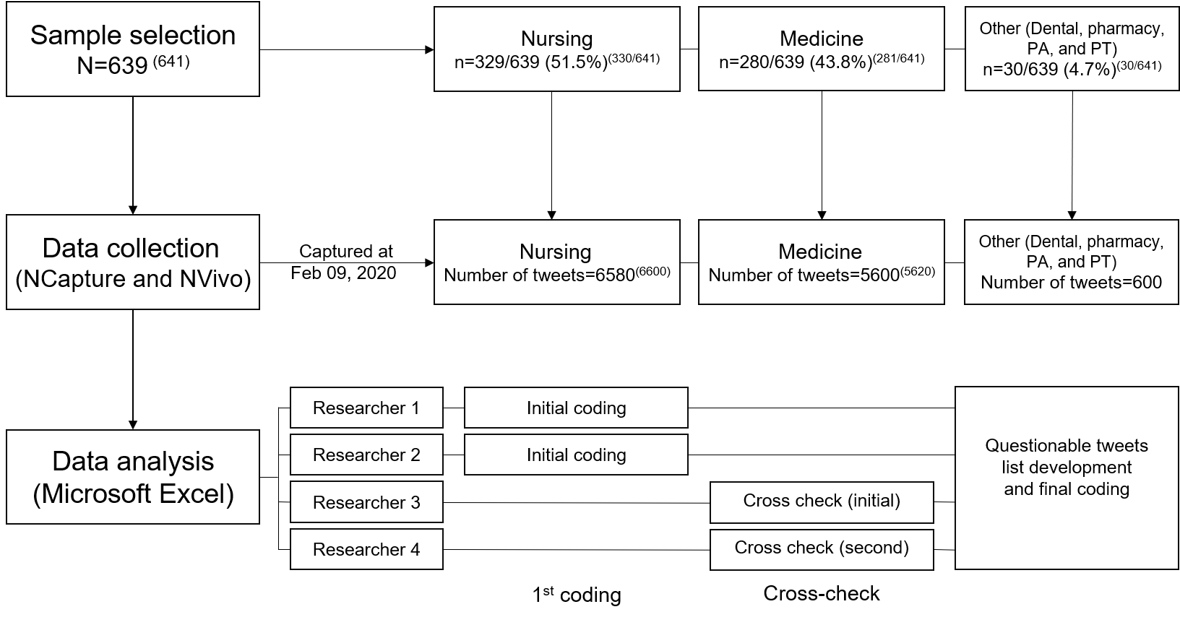
User account selection and data analysis process. PA: physician assistant; PT: physical therapy. Number of data used for analysis is provided within parenthesis in superscript.

Owing to the floating nature of Twitter [5], the users made changes to their accounts during the data collection period. It was difficult to exclude all ineligible accounts with one screening, so 2 researchers (EC and HJ) independently reviewed each account’s profile and content 3 times. We held regular team meetings, discussed the eligibility of accounts based on the criteria, cross-checked the results, and agreed to create additional cut-off criteria (ie, the number of overall tweets and followers) for the final screening (Figure 1). After multiple screenings of ineligible accounts (eg, deleted, banned, locked, or user graduated during the screening; Textbox 3), we ended with a total of 641 health professions student accounts for qualitative analysis and 639 for quantitative analysis (Figure 2).

**Account exclusion criteria for multiple screening.**

**Account exclusion criteria for first screening (n=2579):**

- Not a health professions student account (eg, school, institution, administrator, organization, commercial, business, research only, and not relevant); uses language other than English; user not in nursing, medicine, physician assistant, physical therapy, dental, and pharmacy fields; and not open to public

**Account exclusion criteria for second screening (n=597):**

- User currently working as a health care professional; unclear user identity; and not open to public

**Account exclusion criteria for third screening (n=298):**

- Less than 100 followers; less than overall 50 tweets; uses language other than English; not a current student account; and not open to public

**Account exclusion criteria for fourth screening (n=2; 40 tweets):**

- Deleted and unable to check profile images

### Data Collection

All tweets from 641 accounts were collected through NCapture [25], a free web browser extension tool that allows users to capture the content of web pages, Twitter, and Facebook to import into NVivo (QSR International Pty Ltd). Owing to the uncontrollably large number of total tweets (n=3,415,798), each account’s 20 most recent tweets were purposefully selected and analyzed (N=12,820).

The definition of tweets characterized by incivility (ie, “those written in [an] ill-mannered, disrespectful [way], or containing annoying, derogatory, disruptive, or aggressive remarks”) and various types of a priori codes and their definitions were adopted from the study by De Gagne et al [19] on cyberincivility in Twitter accounts of nurses and nursing students (Table 1). Initially, 2 researchers (EC and HJ) independently examined all 12,820 tweets and identified instances of incivility based on the given definitions. Any unclear tweets were marked as *not sure*. After the initial coding, 2 coders (EC and HJ) had a team meeting and cross-checked the results. Then, a third and fourth coder (SSY and JCD) reviewed all tweets containing inappropriate or potentially objectionable content (cyberincivility) and the tweets marked as *not sure* and provided reasons for their views. When all 4 coders were familiar with the tweets, the team held a meeting to finalize the data set of tweets containing cyberincivility. When the research team identified tweets that fell into gray areas, they considered whether they would post such tweets themselves if they were health professions students and whether they would post them to their Twitter accounts while disclosing their identity; when team members determined that they would not, we categorized those tweets as instances of cyberincivility.

**Table 1:** Codebook used in the study.

Type of incivility	Definition
Profanity^a^	The use of abusive, vulgar, or irreverent words, images, symbols, or acronyms, including wtf, lmfao, or lmao
Product promotion^a^	The promotion to prospective buyers of commercial health or medical products unsupported by evidence through referral to promotional sites or dissemination of information about the product line, brand, or company
Sexually explicit or suggestive^a^	The depiction, description, or suggestion of nudity or sexual content to belittle, degrade, intimidate, humiliate, or harm
Demeaning to patients^a^	Remarks or attitudes toward patients, including body donors, that lack dignity and respect
Name-calling	The use of abusive names to belittle, degrade, intimidate, humiliate, or harm
Rude comments	Comments lacking the respect considered normal in society or conveying contempt with a design to offend, humiliate, or harm
Interprofessional aggression^a^	Expressions of direct/indirect, hostile/subtle, derogatory, or negative attitudes across the health professions
Alcohol and drugs^a^	Depictions of or remarks about health issues such as intoxication that denigrate, condemn, or humiliate a community or its members rather than contributing to safety or education
Violation of privacy and anonymity^b^	Remarks about or images of patients that reveal confidential information or that could be used to identify a patient
Bias and stereotyping references^b^	Prejudicial, discriminatory, or negative remarks or expressions about a culture or a person’s racial, ethnic, religious, gender, or sexual orientation
Intraprofessional aggression^a^	Remarks or expressions of direct/indirect, hostile/subtle, derogatory, or negative attitudes within a given health profession community
Violence^a^	Graphic images or descriptions that glorify violence, suffering, or humiliation or encourage participation
Risky behaviors^a^	Content that encourages, glorifies, or celebrates reckless or unhealthy behaviors, such as speeding, unprotected sex, or hazing that carry a risk of negative results or could lead to loss or harm

^a^Revised definition from the study by De Gagne et al [19].

^b^Revised code from the study by De Gagne et al [19].

### Data Analysis and Rigor

The quantitative data (n=639) were analyzed using SAS version 9.4 (SAS Institute Inc). Descriptive statistics were used to summarize user and account characteristics, including gender; country; type of health discipline; presence of profile images or user descriptions that could be perceived as inappropriate or potentially objectionable; and the number of total tweets, followers, and instances of cyberincivility. We calculated the univariate odds of the presence of cyberincivility for the user and the account characteristics mentioned above with logistic regression.

The qualitative content of tweets containing incivility was analyzed using Microsoft Excel. We performed consensus coding to classify each tweet that could be perceived as inappropriate or potentially objectionable [26]. While using the a priori codes in the findings by De Gagne et al [19], the coding team discussed whether we needed to expand or modify the definition of certain codes or add a new code that could emerge in this study. The team collaborated to create a final set of codes and definitions and consulted a professional editor who provided the team with constructive comments and revisions (Table 1). Then, the coding team independently coded the instances of cyberincivility, cross-checked them, and discussed any discrepancies or disagreements arising among coders to ensure reliability [26]. To ensure the rigor of the qualitative data analysis, all coding team members held regular team meetings during the entire analysis process.

## Results

### Sample Characteristics and Instances of Cyberincivility

A total of 639 accounts were analyzed for quantitative analysis. Of the total 639 accounts, users included 280 (43.8%) medical students, 329 (51.5%) nursing students, and 30 (4.7%) others in 22 countries: 287 (44.9%) from the United States, 197 (30.8%) from the United Kingdom, 104 (16.3%) from unknown countries, and 51 (8.0%) from other 19 countries. The sample comprised primarily female users (489/639, 76.5%) along with 20.8% (133/639) male users and 2.7% (17/639) gender-unknown users. The mean number of followers for each account and the mean number of tweets were 2361.28 (SD 43,443.8) and 5343.50 (SD 10,168.8), respectively. Among the 639 users analyzed for quantitative analysis, 193 (30.20%) tweeted instances of cyberincivility at least once over the 5-week period and had 2.71 instances on average (SD 2.60), with a maximum of 18 and a median of 4. Of the 193 users, 61.66% (119), 32.64% (63), and 5.7% (11) were students in nursing, medicine, and other disciplines, respectively (Table 2).

**Table 2:** Sample characteristics of users (N=639).

Characteristics		Value
Discipline, n (%)		
	Medicine	280 (43.8)
	Nursing	329 (51.5)
	Others	30 (4.7)
Gender, n (%)		
	Female	489 (76.5)
	Male	133 (20.8)
	Unknown	17 (2.7)
Country, n (%)		
	United States	287 (44.9)
	United Kingdom	197 (30.8)
	Others	51 (8.0)
	Unknown	104 (16.3)
Number of followers		
	Mean (SD)	2361.28 (43443.80)
	Median	323.0
Number of tweets		
	Mean (SD)	5343.50 (10168.81)
	Median	1463.0
Instances of cyberincivility^a^, n (%)		
	Absence	446 (69.8)
	Presence	193^a^ (30.2)
Cyberincivility by disciplines (n=193)^a^; n (%)		
	Medicine	63^a^ (32.6)
	Nursing	119 (61.7)
	Others	11 (5.7)

^a^One medical student account was excluded from the quantitative analysis, as some information could not be verified because of account deletion.

The characteristics of accounts with instances of cyberincivility are presented in Table 3, with odds ratios (ORs). Findings from the logistic regression analysis revealed that gender-unknown users were more likely to exhibit instances of cyberincivility than female users (OR 4.9194, 95% CI 1.6086-15.8640). Twitter users with profile pictures that could be perceived as inappropriate or potentially objectionable were more likely to display instances of cyberincivility (OR 3.3484, 95% CI 1.2389-10.0217). Twitter users in nursing were more likely to exhibit instances of cyberincivility than users in medicine (OR 2.1100, 95% CI 1.3009-3.4504). Twitter users from the United States were more likely to display instances of cyberincivility than users from the United Kingdom (OR 3.2172, 95% CI 1.8678-5.6490). Twitter users with fewer followers were more likely to post tweets categorized as instances of cyberincivility (OR 0.5477, 95% CI 0.3033-0.9493). In addition, when they tweeted more often, they were more likely to post cyberincivility (OR 4.6938, 95% CI 3.2626-6.8807). When the number of tweets was equal to 100, if the number of tweets increased by 10%, the odds of the probability of instances of cyberincivility increased to 4.6938 (Table 3).

**Table 3:** Association of Twitter account characteristics with presence of cyberincivility through logistic regression fit.

Characteristics		Estimated coefficient	OR^a^ (95% CI)	*P* value
Gender (reference: female)				
	Male	0.02876	0.9716 (0.5572-1.6702)	.92
	Unknown	1.59319	4.9194 (1.6086-15.8640)	.005
Picture profile (reference: appropriate)				
	Inappropriate or potentially objectionable	1.20850	3.3484 (1.2389-10.0217)	.02
Discipline (reference: medicine)				
	Nursing	0.74669	2.1100 (1.3009-3.4504)	.002
	Others	0.40821	1.5041 (0.6000-3.6218)	.37
Country (reference: United Kingdom)				
	United States	1.16851	3.2172 (1.8678-5.6490)	<.001
	Other	0.87034	2.3877 (0.9871-5.6001)	.048
	Unknown	1.15787	3.1831 (1.7089-5.9744)	<.001
Number of followers this account has		-0.60209	0.5477 (0.3033-0.9493)	.04
Number of tweets issued by the user		1.54624	4.6938 (3.2626-6.8807)	<.001

^a^OR: odds ratio.

### Patterns of Cyberincivility

Over the 5-week period, 3.92% (502/12,820) tweets categorized as instances of cyberincivility were generated by 193 users, comprising 119 nursing (323/502, 64.3%), 64 medicine (155/502, 30.9%), and 10 other health professions students (24/502, 4.8%). Most tweets were collected from the United States (300/502, 59.8%), the United Kingdom (53/502, 10.6%), and Australia (12/502, 2.4%); in addition, 21.5% (108/502) of tweets were collected from unknown locations. A total of 5.8% (29/502) of tweets were collected from 8 other countries that did not have a considerable number of tweets (range 1-10). Of the 502 tweets identified as instances of cyberincivility, 15.5% (78/502) were related to the user’s health profession or school life, and 84.5% (424/502) were related to their personal life. The major categories of the personal life domain were profanity (218/502, 43.4%), product promotion (53/502, 10.6%), and rude comments (42/502, 8.4%). Profanity (37/502, 7.4%) was the most frequent category in the school life domain. The tweets were original posts, responses to other users’ posts, or posts quoted. The frequencies of each code in the personal life and school life domains are shown in Multimedia Appendix 1.

Personal tweets covered a wide range of topics, including entertainment, everyday thoughts and events, relationships, sports, product promotion, service evaluation, and politics. Inappropriate or potentially objectionable tweets in the school life domain were not as prevalent as those in the personal life domain. Tweets in the school life domain that could be perceived as inappropriate or potentially objectionable often expressed students’ frustration or stress with their school (eg, coursework, assignments, grades, exams, and tuition) or aggressively referred to interactions in health care settings or during clinical practice. Some users expressed dissatisfaction with their school’s financial aid office’s expectations or described the stressful nature of the nursing school. A minor number of tweets in the school life domain contained aggressive criticism regarding community health issues or public health policies. One user tweeted about laws that pertained to miscarriage and self-inflicted abortion in what might be interpreted as an opinionated and offensive manner. In tweets categorized as the school life domain, a few users applied school-related hashtags (eg, #medstudenttwitter; #medstudents).

Of the 502 tweets identified as instances of cyberincivility, profanity (255/502, 50.8%) was found most frequently in both the personal life domain (218/502, 43.4%) and the school life domain (37/502, 7.4%). Although the context in which it was used varied, the profanity was generally pointed and direct (eg, expressing frustration with a patient interaction). In some cases, profanity was used to emphasize casual feelings and thoughts. For example, many students used “f**k,” “bit**,” “sh*t,” or the acronym “Lmfao” (“Laughing my f***ing ass off”). Students expressed high levels of dissatisfaction with their elected leaders’ decisions, yet few tweeted profanities at the politicians. Some users tweeted profanity about sports performances or shared and referenced music among other accounts that used profanity. One student tweeted that their progress in school was an “absolute sh*t show.” Sometimes, users used some profanity but censored it with asterisks (ie, F**K). We found 5 accounts that contained profane gestures or words in their profile or header images. Furthermore, there were product promotion-related tweets (60/502, 12.0%) that advertised commercial products, places, websites, or accounts. One tweet referenced traveling around the city and promoted a code for free rides. Some students directly tagged a commercial Twitter account running a money-drawing event and asked for money to pay for their student loan. Some tweets often promoted free show or movie tickets or mobile apps, and a few students shared their customer codes for an extra discount for specific products.

Among 502 tweets coded as instances of cyberincivility, 7.4% (37/502) were of a sexually explicit or suggestive nature, which occurred most frequently in the personal life domain (35/37, 94.6%). In addition, 3.1% (20/639) of users’ profile pictures or images were coded as potentially objectionable because of their sexually suggestive nature to readers or viewers. A few tweets were sexually explicit, including one user’s naked selfies along with an invitation to their personal paid websites (eg, OnlyFans account). Another tweet searched for people with specific sexual fetishes. Most of the sexually explicit and suggestive tweets seemed to have a humorous yet sarcastic or cynical intent. Some tweets portrayed or described excessive alcohol drinking or drug abuse, violent or risky behaviors, or unlawful acts or displayed an image of a weapon. A few users tweeted about biased or stereotyped references to a specific gender, race/ethnicity, culture, or zodiac sign (eg, “Aquarius people are always so rude”). Name-calling (33/502, 6.6%) or tweets meant to belittle, degrade, or humiliate others often occurred between accounts as users argued and expressed disagreement (eg, “idiots”) in response to tweets about current political events or as commentary; these tweets often included derogatory language and were mostly aggressive. For instance, one user referred to a political party in a dismissive manner, and one tweet contained name-calling that expressed opposition to a politician by referring to them as a “toddler” and “a disgrace.” Children and older adults were the targets of 3 tweets that referred to them as disrespectful, stupid, and nasty. Furthermore, 1.4% (7/502) of tweets were coded as demeaning to patients, including tweets about drug seekers observed in the emergency department or tweets that used a mocking tone to describe patients (eg, “they look like the dead”). One user described how they had played with a cadaver’s muscles in an anatomy laboratory.

A proportion of 1.6% of tweets identified as instances of cyberincivility (8/502) exhibited interprofessional (7/502, 1.4%) or intraprofessional (1/502, 0.2%) aggression. Some users tweeted within their own profession (ie, alluded to their work or school) using minor profanity (eg, “Lmao”). Tweets by medical students were dismissive of naturopathic medicine and nurse practitioners: they were mocked in one tweet, and in another tweet, they were deemed not to be a professional. We found 0.8% of those tweets (4/502) that violated privacy and anonymity by providing details of situations and dialogs concerning patients during clinical practice. Although these tweets did not include person-identifiable information, the descriptions provided were sufficiently detailed to allow possible identification by the patients or people involved. Multimedia Appendix 2 summarizes the examples of tweets from each code. All examples have been paraphrased to prevent backtracking and protect privacy while maintaining the original meaning.

## Discussion

### Principal Findings

The purpose of this study is to analyze Twitter content related to cyberincivility among health professions students. Our study sample consisted of a diverse group of students from 22 different countries. Unlike previous studies where a single discipline was included [19,20,27-29], this study explored cyberincivility using a global and multidisciplinary approach.

In our study, 30.2% (193/639) of the sample population engaged in cyberincivility on Twitter at least once over a period of 5 weeks, with an average of 2.71 instances of cyberincivility per user, ranging from 1 to 18 during this period. Regarding a specific discipline, 36.2% (119/329) of nursing students, 22.5% (63/280) of medical students, and 36.7% (11/30) of other health professions students were involved in cyberincivility. In a previous study by De Gagne et al [19], 36.8% of nurses and nursing students posted tweets that could have been perceived as inappropriate or potentially objectionable, which is similar to the findings of this study. The prevalence of cyberincivility among medical students was consistent with a study conducted in the United States [30] in which 21% of medical students self-reported that they had posted profanity, a depiction of intoxication, or sexually suggestive materials on social media. Peer reporting of such content was significantly more frequent than self-reporting [30], which suggests that there may be differing perceptions and opinions of propriety pertaining to social media use. The boundaries of professionalism in cyberspace are likely to be an ongoing topic of discussion among health professionals.

Our study revealed several interesting areas for future research. Gender-unknown users were more likely to engage in cyberincivility compared with users who identified as male or female. A lack of information exists on the relationship between gender identity and cyberincivility; however, gender-unknown users may not be restricted by gender identity [31]. Another interesting finding was that Twitter users with a profile picture that could be perceived as inappropriate were more likely to post potentially objectionable tweets. It has been suggested that as a means of asserting self-presence, a profile picture may provide an emotional statement and a facial image [32]; this is another area that could benefit from further study. It has been noted that social media profiles of medical doctors significantly affect potential patients’ impressions of those doctors’ professionalism [33]; thus, it could be worthwhile to evaluate the potential benefits of profile pictures for building provider-patient relationships and maintaining meaningful connections with the public.

Our findings showed that users from the United Kingdom were more likely to post tweets deemed appropriate than users from the United States and other countries. There have been a few studies on cyberincivility that involved international comparisons. For example, a study of German and Japanese students’ communication on mobile messaging indicated that German students tended to use a direct communication style compared with Japanese students [34]. In our previous study that examined differences in cybercivility among nursing students using cross-country comparisons, we discovered that students from Hong Kong reported lower knowledge of cybercivility compared with respondents from South Korea and the United States [35]. In a study by Kim et al [35], US nursing students reported a lower frequency of cyberincivility experiences compared with students from Hong Kong and South Korea. Although it is difficult to compare our results directly with those from previous studies, they provide further evidence that cultural and societal differences may affect social media communications, thus supporting the development and implementation of proper web-based communication training from a global perspective.

Our findings revealed that Twitter users were more likely to issue potentially problematic content if they had fewer followers. These results may indicate that respondents with many followers may think more about the influence of their tweets and exercise more caution when they post messages. A small number of followers could indicate that followers are closely related to the owner of the account and are therefore not perceived as likely to be influenced or as having dissimilar opinions or social habits. We also noted that Twitter users were more likely to engage in cyberincivility if they posted tweets relatively often. These results are congruent with those of a previous study [19], showing that users who have used Twitter for a longer period may feel more comfortable with the technology and with expressing their opinions freely on even sensitive issues compared with those who have been Twitter users for a shorter period [36].

We found that the largest categories of cyberincivility were profanity and product promotion, which is consistent with the findings of a previous study [19]. Furthermore, we noticed several aggressive or biased comments toward other users, politicians, or specific groups of people. Profanity was reported to be the second most frequent unprofessional content in a study by Kitsis et al [30], which analyzed medical students’ and faculty members’ perceptions of unprofessional content posted on their social networking platforms. Our study showed that students often added minor profane abbreviations (eg, f**k and Lmao) to create an intimate and informal atmosphere to the content of their tweets; however, some students used profanities to show their aggression and offensive opinions toward other users, which could result in fostering similar hostility or rude behavior in their followers. According to negative behavioral contagion models, rudeness is like a cold, and this behavior can be easily activated in social networking and spread easily by any user [37]. In a study by Ryan et al [38] that examined public perspectives on digital professionalism in nursing, participants perceived profanities used generally or against individuals or groups as unacceptable and unprofessional. Such tweets have been reported as rude, disrespectful, and unprofessional in other studies of cybercivility by health professionals and students [19,39].

Although we found relatively few instances of cyberincivility in school-related tweets, their content is worth discussing. We found tweets that included demeaning comments toward specific patient groups or vulnerable populations, including children and older adults, or interprofessional or intraprofessional aggression, such as content that degraded other health professionals. For example, one medical student posted that patients should be treated by physicians rather than by advanced practice registered nurses. In a study by Kitsis et al [30], medical students and faculty perceived social media content as unprofessional if it contained derogatory remarks toward certain patient groups (ie, Medicaid patients) or negative comments about work stress, colleagues, and patients. Similarly, Kim et al [40] studied Korean clinical nurses’ experiences of cyberincivility, including a lack of respect and morality within health professions. They suggested that interprofessional or intraprofessional aggression in online spaces could occur when health care professionals lacked an understanding of the roles of workers in other occupations or when users were tired from work and lost control of their emotions [40]. Researchers have also highlighted that experiences of interprofessional or intraprofessional aggression in cyberspace can increase the workload and stress of health professionals by generating mistrust and reducing teamwork [30,40]. The content of health professions students’ tweets in our study reflects their perceptions, beliefs, and values, and it is possible that their communication with colleagues may indicate a lack of respect and understanding of other occupations. These findings reinforce the need to teach digital professionalism to cultivate respect from students for their peers, colleagues, and patients. The structure of social norms in digital professionalism is complex and evolves based on changing social and individual norms, values, attitudes, beliefs, and context [38]; therefore, instructional materials should include socially and culturally appropriate content and input by individuals from diverse backgrounds.

Although our data did not show many cases of cyberincivility related to privacy violation, several studies have reported social media content that could expose patients’ personal information and invade their privacy [18]. Student disclosure of information about themselves and others (eg, patients or other health care providers) can lead to unexpected consequences. Ahmed et al [18] analyzed 754 tweets issued by doctors, nurses, and other professionals with a hashtag #ShareAStoryInOneTweet containing disclosures about others (eg, patients and colleagues). The content of those tweets included patients’ age, name, specific time frames, clinical images, information about vulnerable groups of patients, and descriptions of direct patient care. Only 2 tweets (0.3%) included the patients’ consent to share the story or information. The authors reported that a considerable number of the tweets are likely to be identifiable by patients or their acquaintances. Their study indicates that sharing clinical stories on the web, including fragmented information, is highly problematic as it can lead to recognition and identification [18] and that health professions students have a clear need for guidelines for safe and professional use of social networking sites [41].

The ubiquitous nature and advanced algorithms of social media allow fast and easy connection with others [42], but this characteristic can blur the line between health professionals (including students) and the public as well as between health care providers’ private and professional lives [42-45]. There is a growing concern about the line between health care providers' privacy and professionalism. Users’ personal information can be easily found through various sources in social networking platforms, including their profile images, everyday narratives, photos taken at work or home, and accounts that they follow or interest groups to which they belong [21,46,47]. Digital footprints, traces that users leave behind on the internet, are archived and can be rediscovered through a simple search [18]. For example, the recent *medbikini* controversy has provoked heated discussion of the standards of digital professionalism after authors of a now-retracted article published in the *Journal of Vascular Surgery* [48] created fake accounts on Facebook, Twitter, and Instagram to analyze the personal posts of graduating vascular surgery trainees for *potentially unprofessional* content, such as pictures of users wearing bikinis or drinking alcohol while off duty.

Researchers, educators, and regulators in health professions have been concerned that posts on the web that are perceived as unprofessional could potentially cost public trust and the professional image of health professions [21,47]. Several studies have recommended that health professionals keep their presence on the web safe and secure by separating professional and private accounts or by using the privacy options of their social media accounts [38,47,49]. Kouri et al [49] argued that health professionals cannot be *general users* of social networking platforms because their identity makes any information or content they post appear reliable and trustworthy, an argument disputed by the professional backlash to the retracted *medbikini* article [48]. Health professions are organized around specialized knowledge in addition to an ethos of duty and service. Historically, these professions have secured autonomy and prominence in the society by adopting codes of ethics and, ultimately, codes of behavior [50]. As social media will most likely continue to provide an important forum for health professions education and social discourse, the growing diversity of thoughts and perspectives about social responsibility and professional ethics should inform cybercivility training for all health professions students.

### Limitations

This study is not without its limitations. First, our study was retrospective and observational and included a sample of accounts during the study period. We analyzed only 20 most recent tweets from each account, which may have skewed the findings. As a logistical challenge, Twitter users frequently change their accounts (eg, lock, ban, delete, or change user IDs) or delete their tweets, so several potential user accounts and tweets were excluded during the data collection phases. We were also solely dependent on the users’ self-reported identification on their user descriptions. If they profiled themselves as health professions students and yet did not appear to be students, our ability to validate their student status was limited. Another possibility of sampling bias relates to our sample primarily consisting of nursing and medical students, with less than 5% of other health professions students (ie, dentistry, pharmacy, physical assistant, and physical therapy) being included. Future studies may explore ways to capture more diverse health professions students.

Second, our study was constrained by time limitations. The content of tweets may vary according to the time frame of the postings. In our case, we completed data collection in February 2020 when the global COVID-19 pandemic was not yet widespread, the Black Lives Matter social justice movement in the United States that followed the death of George Floyd had not commenced, and the August 2020 publication that inspired the *medbikini* issue in the medical profession had not occurred. As social networks respond rapidly to sociocultural and political contexts, these global events and social arguments might have had a significant impact on our results had the data been collected several months later.

Finally, and perhaps most importantly, we are not exempt from researchers’ confirmation bias and cultural bias. Cyberincivility is an emotionally charged social issue that can lead researchers to make interpretations or seek evidence to confirm or support their preconceptions. To minimize such biases, we implemented multiple team meetings during the course of the study, as we identified and analyzed instances of cyberincivility and engaged in open discussions as to why those tweets were potentially problematic. This process was both difficult and beneficial because our team members were of diverse backgrounds and generations, and professional standards are affected by individual experience, culture, generation, life history, and social ambiance. Although it was challenging to measure interrater reliability, the rigor of the study was maintained through deep and insightful team discussions, immersion in data, and a dedicated commitment to limit conflicts arising from cultural or implicit biases [51].

### Future Implications

Work environments that practice professional behavior are safer, more productive, and healthier [52]. Unprofessional behavior has been linked to burnout, absenteeism [53,54], communication breakdowns, increased errors, and decreased performance [54,55]. However, there is still no universal definition of professional behavior. The onset of social media in the last 10 years or more has made it difficult to expand the narrower frameworks of historic codes of ethics [22]. Most major health care professional organizations have published guidelines for the use of social media, and many schools of higher education have them in place as well.

Definitions and rules of professionalism are changeable and have served many functions over time [50]. The relationship between professionals and the public is tenuous, complex, and ever changing; therefore, policies regarding professional codes of behavior, social contracts, and free speech are continuously negotiated. The current and past court cases illustrate the importance of an institution’s ability to define inappropriate off-campus speech. For example, in Keefe vs Adams, the eighth US Circuit Court of Appeals ruled that a nursing student could be expelled for Facebook posts that showed a lack of professionalism [56]. To prevent risks to students and institutions, educators should provide comprehensive and practical guidelines using effective and creative methods (eg, vignettes or simulations) [57,58]. Academic institutions should provide clear policies for students’ social media activities and a safe forum in which all members of the community can constructively discuss controversial issues.

### Conclusions

Cyberincivility is a complex social phenomenon that has an important influence on health professions education. Using the Twitter data mining approach, we analyzed the nature of incivility among health professions students to better understand this concept. Our study supports the existing evidence that cyberincivility is still observed on social media. Twitter is likely to remain a ubiquitous, simple, and convenient tool for communication and education; however, the benefits of using Twitter in health professions education can be maximized only within a culture dedicated to maintaining safe and healthy online communities. Our study shows that there is a continued need to provide students with guidance and training about their online persona and digital professionalism. Our findings have implications for designing evidence-based, intentional, and multidisciplinary cybercivility education rooted in social courtesy, professional ethics, and profound respect for others.

## Appendix

Multimedia Appendix 1 Paraphrased examples of tweets.

Multimedia Appendix 2 Frequencies of each code in the personal and school life domains (n=502).

## Abbreviations

OR: odds ratio
