# Supplementary material for: Analysis of Cyberincivility in Posts by Health Professions Students: Descriptive Twitter Data Mining Study
Source: JMIR Med Educ. 2021 May 13;7(2):e28805. doi: 10.2196/28805 (PMC8160798; doi:10.2196/28805)
Supplement: Multimedia Appendix 1 [file mededu_v7i2e28805_app1.pdf]

Multimedia Appendix 1. Paraphrased examples of tweets.

Type	Domains	
	**Related to Personal life (P)**	**Related to Health Profession or School life (S)**
Profanity	Lmfao, this is f***ing ridiculous.	The new semester began ten days ago, and I'm already annoyed AF (as f***) and I just want to scream.<br>Bit** I got a B in my class, and it makes me f***ing mad.
Product Promotion	I 100% recommend buying this product because I own one and love mine! If you contact me, you can get 20% off! (attached: *product pictures or website link*)<br>You can get free movie tickets by using this link! If you use my link to enter the contest, you and I both get extra entries! (attached: *website link*)	If you are my Twitter follower, you can get 20% off on the new NCLEX test prep and get an electronic gift card! Use the link in my bio.<br>*@XXXX (money-drawing event account ID)* I'm a poor student; please give me money and help me pay off my student loan!
Sexually Explicit or Suggestive	Exercise can make your sex life so much better! (attached: *a half-naked selfie*)<br>I am looking for someone with a foot fetish because my beautiful feet are ready to show and get paid.<br>Watching a music performance with my dad and he says "it sounds like porn"	*None*
Demeaning to Patients	*None*	During anatomy dissection, we had a chance to expose all the eye muscles of the cadaver. It may sound weird, but I enjoyed pulling them repeatedly and making the eye movement as if it were alive.<br>Those skinny people with eating disorders look like the dead. (attached: *retweet writing concerns about false recognition of eating disorder*)
Name-calling	*@Twitter ID* You are so stupid and disgraceful. You are an idiot	*@Twitter ID* I don't want to waste my time with silly [*political preference*] people who do have no medical knowledge. Now I need to go into clinical, which is much more important than making you idiots angry.
Rude Comments	I hate kids because they are so annoying. They get sick easily and make me snuggle them (and make me feel guilty if I don't). And they thank me by spreading their germs. They are so nasty, just like walking Petri dishes.	Pro-life people should keep their teratoma because it is basically a living thing.
Interprofessional Aggression	*None*	Recently I went to urgent care for a headache. I got treated by a nurse practitioner who didn't even have basic

		medical knowledge and didn't perform any necessary physical examinations.<br>I f***ing hate health care people who live on social media pages.<br>Seeing that he was using such stupid words, he must be a (*other health professions*).
Alcohol & Drugs	Hey, it's a special day and I'm super drunk now. I hope I'm surrounded by nice men and women and make out with them. #DrunkTweets (attached: *a selfie holding a bottle of alcoholic beverage*)	*None*
Violation of Privacy and Anonymity	*None*	I met a dad and his son when I was doing my clinical rotation in the ER. The dad kept asking me to do [*specific situations that can reveal the identity of the patient/caregiver or be recognized by the people who know the patient/caregiver*], which was so weird.
Bias and Stereotyping References	Some people eat grasshoppers, ewww, so gross.<br>N***** are so complicated and hard to understand.<br>All men/women are so stupid. They are all trash.<br>Boomers in my workplace have a meltdown because they can't use and understand technology. They just shout at young people and ask young people to bring them drinks.	*None*
Intraprofessional Aggression	*None*	My teaching assistant had the audacity to give me a bad grade on a quiz for spelling the name of a microbe wrong when he can't even spell simple words. So ridiculous.
Violence	(attached: *a picture of a gun in the video game*)	*None*
Risky Behaviors	The fact that I really went to jail last night is crazy and I was freaking miserable there. And no one answered their phones. It was freaking crazy	If you want to lose a lot of weight in a short period of time, the combination of methamphetamine and cocaine worked for people I met in the ER, but you will see the walls talking to you.
