# Supplementary figures and images for: Analysis of Cyberincivility in Posts by Health Professions Students: Descriptive Twitter Data Mining Study
Source: JMIR Med Educ. 2021 May 13;7(2):e28805. doi: 10.2196/28805 (PMC8160798; doi:10.2196/28805)

**Multimedia Appendix 2.** Frequencies of each code in the personal and school life domains (n=502).

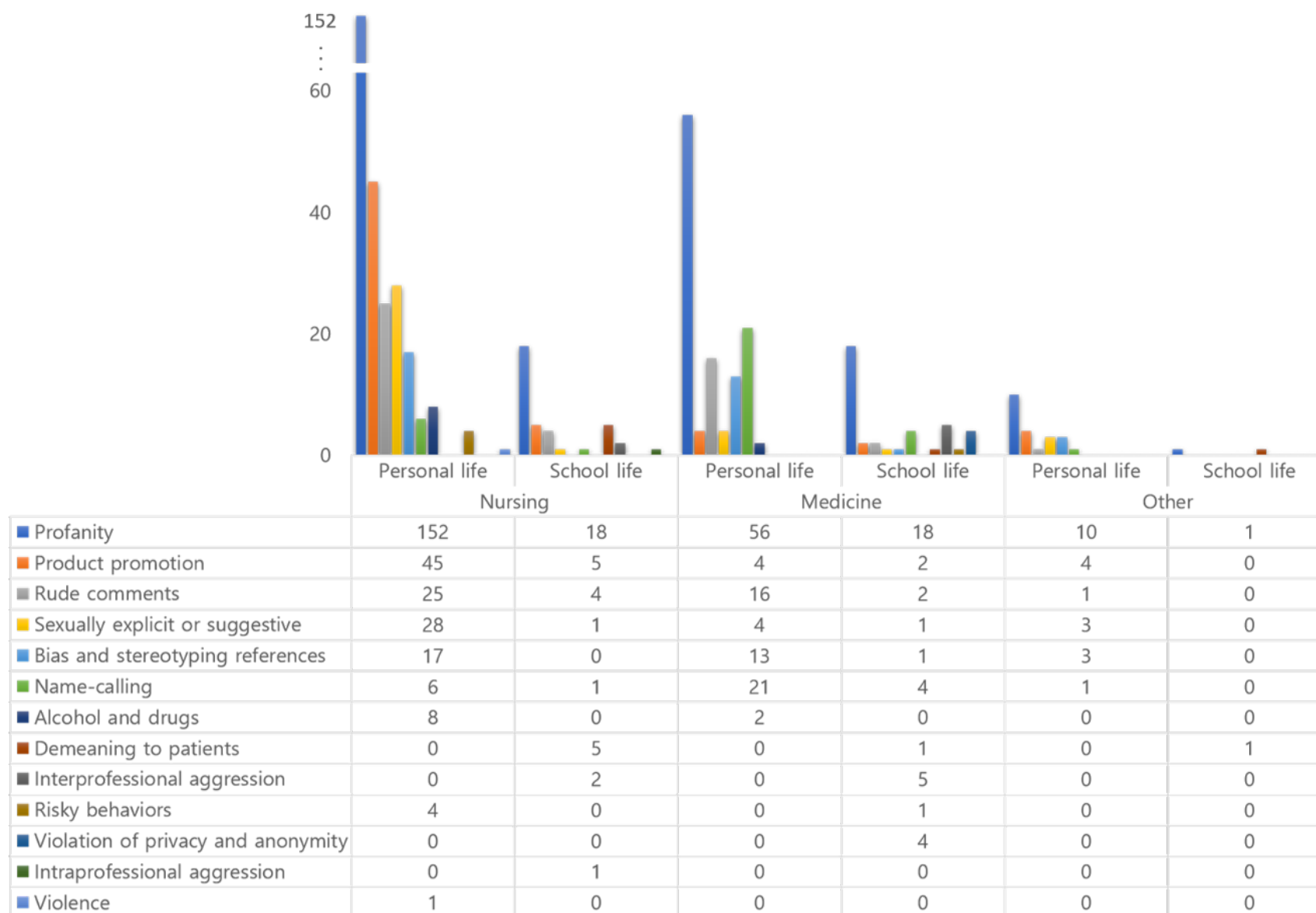

Supplement: Multimedia Appendix 2 [file mededu_v7i2e28805_app2.pdf]
